# Correlation Analysis of Cocoa Consumption Data with Worldwide Incidence Rates of Testicular Cancer and Hypospadias

**DOI:** 10.3390/ijerph6020578

**Published:** 2009-02-05

**Authors:** Fabrizio Giannandrea

**Affiliations:** Industrial and Environmental Hygiene Unit, Department of Animal and Human Biology, University “Sapienza”, Rome, Italy. E-mail: fabrizio.giannandrea@uniroma1.it; Tel.: +39-06-49912682; Fax: +39-06-49912771

**Keywords:** Male reproductive diseases, testicular cancer, hypospadias, cocoa consumption

## Abstract

The underlying reasons for the increasing occurrence of male reproductive diseases (MRD) such as hypospadias, cryptorchidism, and testicular cancer (TC) over the last decades are still unknown. It has been hypothesized that the risk of MRD is determined *in utero* and that pregnancy dietary intake could also affect MRD risk in the offspring. Various studies in animals reported that cocoa and theobromine, the main stimulant of cocoa, exert toxic effects on the testis, inducing testicular atrophy and impaired sperm quality. A correlation analysis was conducted to examine the possible role of cocoa consumption on the occurrence of selected MRD during the prenatal and early life period of cases. The incidence rates between 1998–2002 of TC in 18 countries obtained from Cancer Incidence in Five Continents were correlated with the average per-capita consumption of cocoa (kg/capita/year) (FAOSTAT-Database) in these countries from 1965 to 1980, i.e. the period corresponding to the early life of TC cases. In order to test the above correlation in the case of hypospadias, the mean prevalence at birth in 20 countries (1999–2003) with average per-capita consumption of cocoa in these countries in the same period corresponding to pregnancy were used. The consumption of cocoa in the period 1965–80, was most closely correlated with the incidence of TC in young adults (r=0.859; p<0.001). An analogous significant correlation was also observed between early cocoa consumption and the prevalence rates of hypospadias in the period 1999–2003 (r=0.760; p<0.001). Although the ecological approach used in this study cannot provide an answer on the causal relationship between consumption of cocoa in early life and TC and hypospadias, the results are suggestive and indicate the need of further analytic studies to investigate the role of individual exposure to cocoa, particularly during the prenatal and in early life of the patients.

## 1. Introduction

Male reproductive tract diseases (MRD) such as hypospadias, cryptorchidism, and testicular cancer (TC) have been increasing for at least 40 years in the developed countries. In particular, TC is currently the most diagnosed malignancy in men aged 20–34 years and its incidence in some countries has risen 3-fold over the last decades [1–3]. These male developmental disorders have been proposed to comprise a common underlying syndrome with a common aetiology resulting from the disruption of embryonic programming and gonadal development during fetal life, termed Testicular Dysgenesis Syndrome (TDS) [4]. A hormonal etiology most likely underlies this syndrome, although it is believed to have more than one cause, possibly including nutrition and high calorie intake during early life [4–6].

The pathogenetic role of nutrition originated from several epidemiological observations of constantly increasing incidence of TC since the beginning of the 20th century with the only major interruption in this trend occurring for men born during World War II or immediately thereafter, when the daily energy intake had been dramatically reduced [6]; moreover, it has been proven that poor and nutritionally underprivileged populations such as African, including U.S. blacks, and Asian populations experience a lower risk of MRD than more affluent people [6].

The increasing occurrence of MRD over time in developed countries might be consistent with increases in the consumption of some sweet foods, such as cocoa. The *per-capita* intakes of cocoa, in fact, have been increasing since at least 1961 year in most developed countries [8]. For example, in Denmark cocoa consumption more than doubled in the period 1961–2004. Similar trends have been observed in other European countries [8,9].

In addition, several studies have reported that cocoa and its main stimulant theobromine exert toxic effects on the testis, inducing testicular atrophy accompanied by aspermatogenesis or oligospermatogenesis and that even low doses of cocoa impair sperm quality [10–13].

In human cells *in vitro*, theobromine induced sister chromatid exchange and chromosomal breaks. In cultured mammalian cells, it induced gene mutations and sister chromatid exchange [41]. Theobromine was also found to produce a higher and more linear rate of sister chromatid exchange damage than caffeine [10,14]. In addition, progeny from the mice fed chocolate at doses similar to those consumed by “high” consumption human populations, presented considerable morphometric abnormalities in the kidney structure, with the lower number of glomeruli per mm^2^ and their increased diameter [15].

As dietary patterns of cocoa consumption are extremely difficult to investigate retrospectively in humans, a correlation analysis was conducted to examine the possible role of cocoa consumption on the occurrence of selected male reproductive disorders during the prenatal and early life period of cases.

## 2. Methods

The incidence rates of TC between 1998–2002 in 18 countries (Denmark, Finland, Norway, Sweden, U.K., Italy, Spain, France, Switzerland, Germany, Poland, Netherland, USA, China, India, Australia, Canada, Japan) obtained from Cancer Incidence in Five Continents (Source: International Agency for Research on Cancer - IARC) were correlated with the average per-capita consumption of cocoa (Source: Food and Agriculture Organization - FAO) in these countries from 1965 to 1980, i.e. the period corresponding to the early life of TC cases [9,16].

In this analysis, data from 18 countries, for which both truncated age-adjusted cancer incidence rates for 20–34-year-olds and detailed cocoa consumption data (FAOSTAT Database) in the period corresponding to the gestation and infancy of the cases (1965–1980) were available, were used. Incidence rates for 20–34-year-olds were chosen because most cases of testicular cancer occur in this age group.

Several countries reported data to the IARC from only one local cancer registry that was assumed to represent the incidence rate for the whole country (e.g. Canada, Denmark, Finland, The Netherlands, Norway and Sweden). Where multiple registries from one country were available, the average value of the age-adjusted incidence rates was employed as a representative rate for the country (e.g. Australia, China, France, Germany, India, Italy, Japan, Peru, Poland, Spain, Switzerland and UK). Since in the U.S. more than one cancer registry is operating, the registry for white people by the SEER program was chosen as a representative value for the U.S [16].

The cocoa consumption data (kg/*capita*/year) from 1965 to 80 available from the FAOSTAT Database were used [9].

*The International Clearinghouse for Birth Defects Monitoring Systems* (ICBDMS), a nongovernmental organization of the World Health Organization, collects data on selected birth defects from countries that have actively functioning registries. Hypospadias is included in this set of birth defects [17].

In order to test the above correlation in the case of hypospadias, the mean prevalence at birth in 20 countries (1999–2003 from ICBDMS Database) with average per-capita consumption of cocoa (FAOSTAT-Database) in these countries in the same period corresponding to pregnancy were used.

Hypospadias was specifically chosen trying to avoid the risk of possible underestimation reported for other congenital defects of the male reproductive tract such as chryptorchidism, and because the available data on this condition are rather accurate [18].

All of the data were analyzed by SPSS-13 (SPSS Institute, Chicago). The 0.05 level of probability was used as the criterion for statistical significance. The Pearson product-moment correlation coefficients (r values) between the incidence of TC in 18 countries and the per-capita consumption of cocoa were examined. The same procedure was followed in the case of hypospadias.

## 3. Results

**Figure 1.**
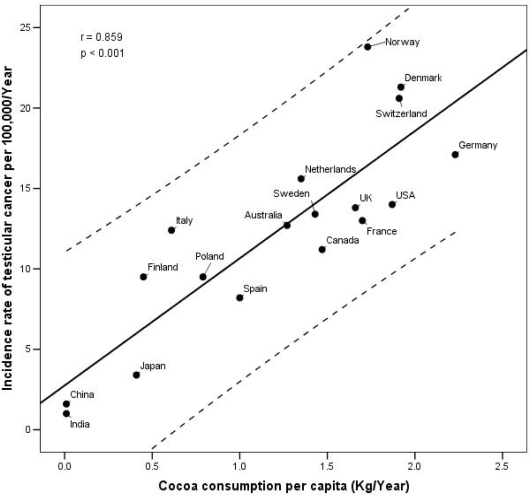
Correlation between TC incidence rates for 20–34 years-olds (1998–2002) and per capita cocoa consumption (1965–1980) in 18 countries.

The 1998–2002 truncated age-adjusted incidence rates for testicular cancer in the 20–34 age group varied greatly from one country to another; Norway had the highest rate at 23.8/100,000, followed by Denmark (21.3), Switzerland (20.6) and Germany (17.1) (Figure 1). The lowest rate was found in India (1.0) and China (1.6). Consumption of cocoa during the corresponding period of pregnancy or infancy seems to follow similar patterns to TC incidence rates of adult life. In fact, the highest consumption (kg/*capita*/year) of cocoa was registered for the Nordic countries such as Denmark (1.92), Germany (2.23) and Norway (1.73) together with Switzerland (1.91), while the lowest in India (0.01) and China (0.01).

The correlation coefficients for the association between the incidence rate of TC and the cocoa intake in 18 countries confirm these preliminary observations. The consumption of cocoa in the period 1965–80, was most closely correlated with the incidence of TC in young adults (r=0.859; p<0.001). An analogous significant correlation was also observed between early cocoa consumption and the prevalence rates of hypospadias in the period 1999–2003 (r=0.760; p<0.001).

**Figure 2.**
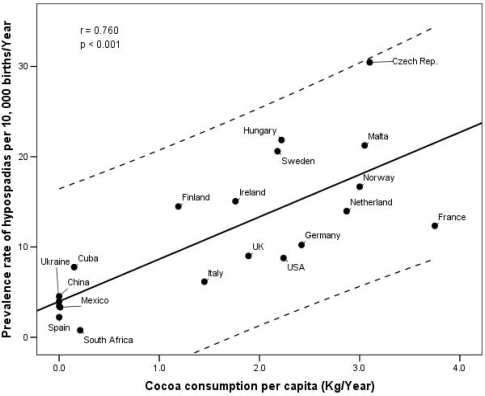
Correlation between prevalence rate of hypospadias (1999–2003) and per capita cocoa consumption in 20 countries in the same period corresponding to pregnancy.

Figures 1 and 2 show the correlation between these two diseases and the consumption of cocoa in the early life period of the cases.

**Figure 3.**
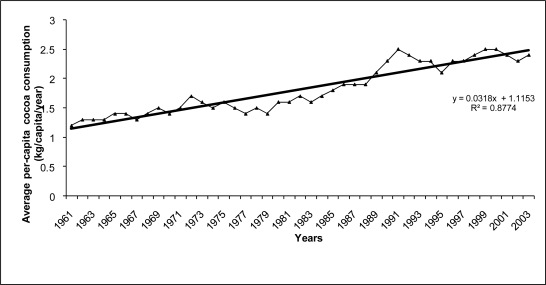
Trend in average per-capita consumption of cocoa (1961–2003) in nine European countries (Denmark, Finland, Norway, Sweden, U.K., Italy, Spain, France and Switzerland)

Analysing cocoa beans consumption trends in selected developed countries in the last 40 years, we observed a steady increase of 1% per year, that seems to be consistent with the parallel increases in the incidence of TC and other male reproductive diseases registered in the same period [3]. Figure 3 shows a linear trend (R^2^ = 0.877) in average per-capita cocoa consumption in nine European countries (Denmark, Finland, Norway, Sweden, U.K., Italy, Spain, France and Switzerland) from 1961 to 2003.

An analysis was also undertaken to examine whether the correlation of TC with cocoa intake was stronger for the prenatal period, than for a period of time in later life, using a method developed by Paulozzi [19].

The finding was that the association between TC incidence rates (1998–2002) among men in their 20s, 30s and 40s is stronger with cocoa consumption in their prenatal period (1965–1980) than the association with cocoa consumption in the 1990s (adulthood) (Table 1). This, together with the high correlation of cocoa consumption with hypospadias prevalence, suggests that the critical exposure period might be closer to birth rather than in later life.

## 4. Discussion

This correlation analysis suggests that cocoa consumption during early life might be correlated to both TC incidence among young men aged 20–34 years and hypospadias, a reproductive congenital defects supposed to underline the same pathogenetic mechanism of TC.

In particular, the increasing incidence of TC over time in developed countries is consistent with increases in the consumption of cocoa. The intake of some of the main sweet ingredients, in fact, such as cocoa and sweeteners increased significantly during the last 45 years in developed countries [8,9]. Over the years 1961–2004 cocoa consumption overall in developed countries grew at an average rate of 1 % per year [9]. For example, in Denmark cocoa consumption more than doubled in the period 1961–2004. Similar trends have been observed in other European countries [8,9]. The magnitude of these increases is similar to those noted for incidence rates of testicular cancer and other MRD. The reduced risk for testicular cancer for the cohorts of Danish, Norwegian, Swedish and Japanese men born during World War II is also consistent with a greatly reduced supply of sweet foods and cocoa during the war.

Data from food balance sheets (FAOSTAT) indicate that the consumption of cocoa in Denmark, where TC and hypospadias rates are elevated, is among the highest in the world and is more than three times that in Finland, where MRD rates are rather low [9].

Is there any evidence that this association may be causal?

Cocoa powder is a complex substance containing several biologically active compounds, including theobromine, caffeine, serotonin, phenylethylamine and cannabinoid-like fatty acids [11].

Various studies reported that theobromine, the main stimulant of cocoa, exerts toxic effects on the testis, inducing testicular atrophy accompanied by aspermatogenesis or oligospermatogenesis and that even low doses of cocoa impair sperm quality [10–14].

Friedman *et al.* reported that feeding theobromine to male Osborne-Mendel rats at a dietary level of 0.5% for 64 weeks resulted in severe testicular atrophy in 94% of animals, with aspermatogenesis in 82% [20]. The results were confirmed in another strain of rats; following 19 weeks of feeding theobromine, all rats showed atrophy, and 79% had aspermatogenesis [10].

Tarka *et al.* found that feeding theobromine at levels of 0.2–1.0 % in the diet (90–140 to 500–600 mg/kg bw per day) for a period of 28 days to rats produced severe testicular atrophy at the 0.8% level and seminiferous tubular-cell degenerationat the 0.6% level [13]. These authors also studied the potential reversibility of this phenomenon by feeding proven breeder male Sprague-Dawley Rats 0.2, 0.6 or 0.8% theobromine (88, 244 or 334 mg/kg bw per day, respectively) for 49 days, performing unilateral orchiectomy at that time and allowing rats to recover on a theobromine-free diet for an additional 49 days. Histologically, the effects at the two highest dose levels were largely irreversible [13].

Funabashi *et al.* administered orally theobromine to male Sprague-Dawley rats at dose levels of 250 and 500 mg/kg for 2 weeks starting at the age of 6 or 8 weeks, and for 4 weeks from the age of 6 weeks [21]. Histopathological examination of reproductive organs revealed toxic findings in the testis such as degeneration/necrosis and desquamation of spermatids and spermatocytes, vacuolization of seminiferous tubules, and multinucleated giant cell formation at 500 mg/kg after 2 weeks of dosing at both ages, and at 250 and 500 mg/kg after 4 weeks of dosing [21].

There was also genetic effects of theobromine in animals. *In vivo*, theobromine induced sister chromatid exchange and micronuclei in the bone marrow of Chinese hamsters. In human cells *in vitro*, theobromine induced sister chromatid exchange and chromosomal breaks. In cultured mammalian cells, it induced gene mutations and sister chromatid exchange [10,14]. In particular, theobromine was found to produce a higher and more linear rate of sister chromatid exchange damage than caffeine [10,14]. In addition, progeny from the mice fed chocolate presented considerable morphometric abnormalities in the kidney structure, with the lower number of glomeruli per mm^2^ and their increased diameter [15].

These findings from animal studies can be compared with the only human study on exposure to theobromine during pregnancy and early childhood [22]. This study examined the level of theobromine in mother’s milk after ingestion of 113 g (4 ounces) milk chocolate (containing a total of 240 mg of theobromine). Peak theobromine concentrations of 3.7 to 8.2 mg/L were found in all fluids including breast milk at 2 to 3 hour after ingestion of the chocolate [22]. According to the European Food Safety Authority (EFSA), if this amount of chocolate were to be ingested four times a day, it would potentially lead to an exposure of the breast fed infant of about 10 mg theobromine/day (corresponding to 1–2 mg/kg b.w.) [23]. Such an exposure might result in pharmacologically active theobromine levels since newborn babies and infants have very low CYP1A2 activity and would metabolise theobromine much more slowly than adults [23].

An alternative explanation of the relationship between cocoa consumption and occurrence of MRD might be that consumption of cocoa and chocolate-containing confectionery could be related to the hormonal equilibrium of the mother during pregnancy and of the infant in the development of MRD in later life. Consumption of energy-dense foods during pregnancy or childhood could negatively effect the balance between free and bound estrogens. The foetus could be exposed to increased estrogen levels through the availability of the high maternal estradiol levels occurring during pregnancy. Bioavailability of estradiol during pregnancy is mainly regulated by the levels of sex hormone-binding globulin (SHBG), whose most important endogenous suppressor is insulin [24]. It is well-established that sweet and cocoa consumption is associated with increased levels of insulin. In fact, though cocoa and chocolate-containing confectionery elicits only a low glycemic response due to its poor sugar content, insulin responses to chocolate is disproportionately high, being almost 50–60 % higher than its glucose score [25].

Fung *et al.* found recently that a Western pattern, which represents a higher intakes of sweets and desserts, was associated with a higher level of estradiol and lower concentration of SHBG in women [26].

In particular, it was observed that pregnancy intake of sweets was significantly related to the reduction of serum sex hormone-binding globulin (SHBG) at the 16th gestational week [27]. The exposure to free estrogens during pregnancy was suggested to increase the risk of MRD in the offspring later in life [4,5].

Finally, cocoa beans are some of the principal dietary sources of ochratoxin A, a mycotoxin that causes adducts in testicular DNA of rats and is an established cause of liver and kidney tumors in rodents and is a testicular toxicant [28,29]. In addition, there is some evidence that ochratoxin might be a cause of TC [29]. Surprisingly, there were no previous studies on the cocoa consumption in the development of MRD in humans, whereas toxic effects on male reproduction in animals are established.

We chose specifically hypospadias and TC trying to avoid the risk of possible underestimation reported for other congenital defects of the male reproductive tract, such as chryptorchidism, and because the available data on these conditions are rather accurate. The ecological approach used in this analysis has well known limitations. For example, it is not to be excluded that cocoa consumption may be simply a surrogate marker of affluence or other environmental factor. The data on the prevalence of hypospadias could be incomplete or non uniform, while the food consumption data may not apply to the cases under study. In fact, ecological studies correlating prevalence and/or incidence rates of a disease with the dietary practices in various geographical areas can only generate hypotheses for further studies.

Food consumption data provided by the FAO present other types of limitations, including differences in classification and definition of food items, completeness, and spatial and temporal variability and could not be adjusted for age, gender and other possible confounding factors, such as alcohol intake.

In addition, FAO consumption data may be particularly incomplete in developing countries such as India and China. However, we separated the analysis excluding these two countries and found the same results.

These findings, therefore, suggest that the associations found may not be spurious, and might deserve further studies of hypospadias and TC at the individual level to investigate the role of dietary intake of cocoa.

## 5. Conclusions

Our findings suggest that exposure to cocoa during prenatal life or childhood may be associated to the risk of both hypospadias and testicular cancer in the offspring. The association between MRD and consumption of cocoa has been discussed in relation to reproductive toxicity of cocoa theobromine. However, it is not to be excluded that an increase in insulin production, the inhibition of serum SHBG and consequent increase of free circulating oestrogen levels could have a direct role on the occurrence of MRD. Correlation studies on the association of diseases rates with the dietary practices in various geographical areas can only generate hypotheses for further studies. It is probable that the data sets from developing countries are less complete than those from developed countries because of problems with under-diagnosis. Nevertheless, the overall association is convincing, plausible and at least may serve to stimulate further epidemiological and experimental studies.

At present, there are no other epidemiologic studies examining specifically the association between TC and hypospadias and consumption of cocoa. The evidence examined here suggests that, future analytic studies at the individual level of TC hypospadias and other reproductive disease of the male reproductive system, should investigate the role of dietary intake of cocoa and sweet foods, particularly during the prenatal and in early life of the patients.

## Figures and Tables

**Table 1.** Correlation coefficients between the incidence of TC by age group in 18 countries (1998–2002) and cocoa consumption in two periods: in prenatal life (1965–1980) and in adulthood (1990–1995).

Age group (years)	Years of cocoa intake	
	1965–1980	1990–1995

0–19	0.606	0.260
20–29	0.824	0.529
30–39	0.897	0.670
40–49	0.912	0.725
All ages	0.884	0.631
